# Outcomes of allogeneic ocular surface stem cell transplantation

**DOI:** 10.3389/fopht.2026.1836045

**Published:** 2026-06-11

**Authors:** Albert Y. Cheung, Natalia Quiroz-Casian, Enrica Sarnicola, Gautam C. Ramanathan, Siddharth Nath, Edward J. Holland

**Affiliations:** 1 Virginia Eye Consultants, Norfolk, VA, United States; 2 Department of Ophthalmology, Eastern Virginia Medical School, Norfolk, VA, United States; 3 Cincinnati Eye Institute, Cincinnati, OH, United States; 4 Clinica degli Occhi Sarnicola, Grosseto, Italy; 5 Department of Ophthalmology and Visual Sciences, McGill University, Montréal, QC, Canada; 6 Department of Ophthalmology, University of Cincinnati, Cincinnati, OH, United States

**Keywords:** allogeneic, living-related conjunctival limbal allograft (lr-CLAL), cultivated limbal stem cell transplantation (CLET), keratolimbal allograft (KLAL), limbal stem cell deficiency (LSCD), ocular surface stem cell transplantation (OSST), simple limbal epithelial transplantation (SLET), systemic immunosuppression

## Abstract

Allogeneic ocular surface stem cell transplantation (OSST) is an established therapeutic approach for limbal stem cell deficiency, utilizing techniques such as keratolimbal allograft (KLAL), living-related conjunctival limbal allograft (lr-CLAL), allogeneic cultivated limbal epithelial transplantation (allo-CLET), and allogeneic simple limbal epithelial transplantation (allo-SLET). This review synthesizes evidence from 35 studies encompassing 1, 268 eyes with at least 24 months of follow-up to evaluate intermediate- and long-term outcomes. Overall, OSST is associated with improvement in visual acuity and restoration of ocular surface stability, although success rates vary widely by technique, ranging from 13% to 87%. Outcomes appear to be influenced by the intensity of systemic immunosuppression, with triple-agent regimens demonstrating higher rates of long-term success compared with single- or dual-agent approaches. Among techniques, lr-CLAL may offer advantages over KLAL due to lower rejection rates. Despite encouraging results, long-term data remain limited for newer approaches such as allo-CLET and allo-SLET. Careful postoperative monitoring and management of complications, including rejection, glaucoma, and microbial keratitis, remain essential to optimize outcomes.

## Introduction

Corneal stem cells are located along the limbus and are responsible for producing healthy corneal epithelium (1). Limbal stem cell deficiency (LSCD) is the result of dysfunctional or decreased stem cells with the subsequent inability to maintain normal corneal epithelial integrity (2). There are a variety of etiologies that can cause LSCD, including traumatic/toxic (e.g. iatrogenic, contact lenses, chemical/thermal injuries), inflammatory (e.g. Stevens Johnson Syndrome [SJS], mucous membrane pemphigoid [MMP], allergic, severe dry eye, infectious), and congenital (e.g. aniridic). Based on the extent of the damaged area to the cornea, LSCD can be divided into partial or total. LSCD is characterized by features including conjunctivalization with invasion of conjunctival goblet cells onto the corneal surface, corneal neovascularization (CNV), epithelial haze, persistent epithelial defects (PED), and ulceration.

There is often concomitant conjunctival deficiency in the setting of LSCD which leads to chronic inflammation, symblepharon formation, forniceal loss, extensive goblet cell loss with mucin deficiency, severe dry eye, and eventually keratinization (3). The conjunctival involvement not only contributes to a severe loss of corneal clarity, impairment of visual function, and chronic pain, but it is also the major factor determining prognosis with the different management options (4).

**Figure 1:**
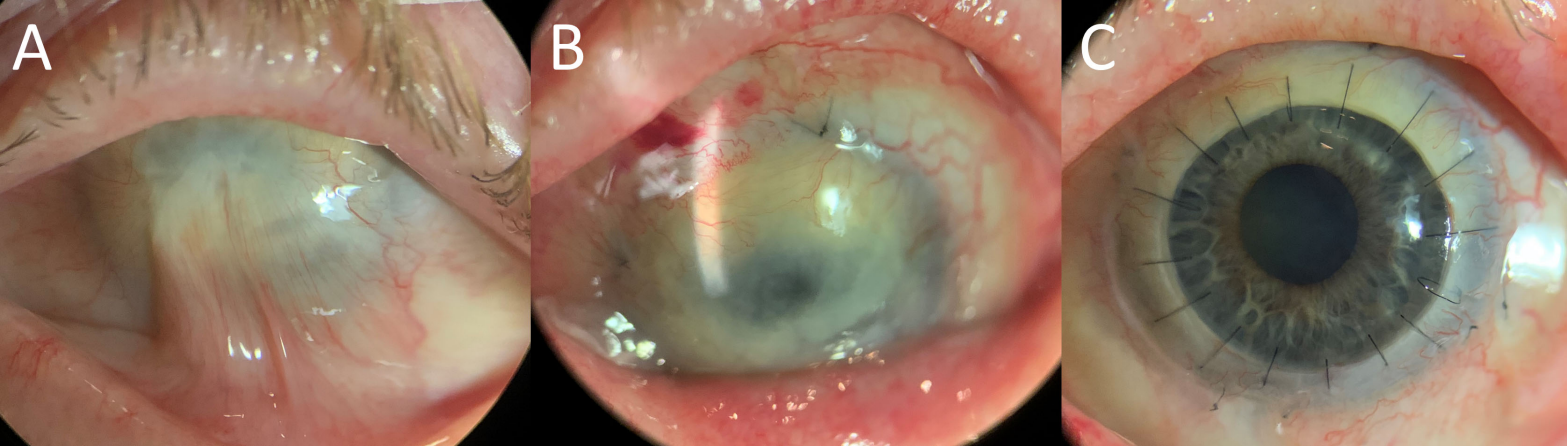
Slit lamp photographs demonstrating management of conjunctival and limbal stem cell deficiency (LSCD) following a remote alkaline chemical injury. **(A)** Demonstrates ocular surface failure, dense inferior symblepharon, corneal neovascularization, scarring, and LSCD. **(B)** Demonstrates a stable ocular surface following a Modified Cincinnati Procedure consisting of combined conjunctival limbal autografts (superior/inferior) and keratolimbal allografts (nasal/temporal). This was followed by a deep anterior lamellar keratoplasty due to corneal scarring **(C)**.

Ocular surface stem cell transplantation (OSST) procedures can provide healthy limbal stem cells and conjunctiva to rehabilitate a damaged ocular surface (5) (**Figure 1**). The surgical technique is selected based on the extent of limbal involvement, degree of conjunctival deficiency, laterality, and clinical condition of the contralateral eye. The most common types of OSST include conjunctival-limbal autograft (CLAU) (6), living-related conjunctival limbal allograft (lr-CLAL) (7, 8), keratolimbal allograft (KLAL) (9), cultivated limbal epithelial transplantation (CLET) (10), simple limbal epithelial transplantation (SLET) (11), cultivated oral mucosal epithelial transplantation (COMET) (12), or combination procedures (13–15). While the details of each surgical technique have previously been described in the literature, the main differences are highlighted in **Table 1**.

**Table 1:** Ocular surface stem cell transplantation methods.

Procedure	Donor	Transplanted tissue	Advantages	Disadvantages
Conjunctival limbal autograft (CLAU)	Fellow eye (autologous)	Limbus + conjunctiva	Provides stem cells and conjunctiva. No SI required.	Only for unilateral injuries
Living-related conjunctival limbal allograft (lr-CLAL)	Living relative eye (allogeneic)	Limbus + conjunctiva	Provides stem cells and conjunctiva. Treats bilateral disease	Requires HLA-matching. SI required
Keratolimbal allograft (KLAL)	Deceased donor eye (allogeneic)	Limbus + cornea	Provides more stem cells. Treats bilateral disease	No conjunctiva provided. SI required
Cultivated limbal epithelial transplantation (CLET)	Fellow, deceased donor, or living relative eye (autologous/allogeneic)	Limbus (*ex vivo* cultivated)	Small amount of donor tissue taken.	Required laboratory, increased costs. No conjunctiva provided. SI required for allo-CLET
Simple limbal epithelial transplantation (SLET)	Fellow, deceased donor, or living relative eye (autologous/allogeneic)	Limbus (*in vivo* expansion)	Small amount of donor tissue taken.	No conjunctiva provided. SI required for allo-SLET
Cultivated oral mucosal epithelial transplantation (COMET)	Autologous oral mucosa	Oral epithelium	No risk to donor eyes. No SI required	Required laboratory, increased costs. No conjunctiva provided.
Cincinnati procedure (lr-CLAL + KLAL)	Living relative eye; deceased donor eye (allogeneic)	Limbus/cornea + conjunctiva	Provides more conjunctiva and stem cells	Requires HLA-matching. SI required
Modified Cincinnati procedure (CLAU + KLAL)	Fellow eye; deceased donor eye (autologous + allogeneic)	Limbus/cornea + conjunctiva	Provides more conjunctiva and stem cells	Cannot treat bilateral disease. SI required
Combined CLAU + lr-CLAL	Fellow eye; living relative eye	Limbus + conjunctiva	Provides more conjunctiva and stem cells	Requires HLA-matching. SI required. Cannot treat bilateral disease

HLA, human leukocyte antigen; SI, systemic immunosuppression.

In the setting of bilateral LSCD, severe unilateral LSCD (especially with coexisting conjunctival deficiency), or when there is not a suitable contralateral eye to contribute stem cells, an allogeneic OSST procedure such as lr-CLAL, KLAL, allogeneic CLET (allo-CLET), or allogeneic SLET (allo-SLET) may be selected (5). Allografts are obtained either from a living-related donor or a deceased donor. Due to the vascularity of the limbus and presence of antigen presenting cells, patients undergoing allograft procedures typically require adequate immunosuppression to achieve excellent outcomes (16). This paper reviews the intermediate and long-term outcomes of allogeneic OSST surgeries. Although keratoprosthesis and non-limbal based OSST procedures (e.g. oral mucosa, conjunctival) may be an option for bilateral disease, these were outside the scope of this review.

## Materials and methods

A literature search of studies listed in the PubMed, MEDLINE, Scopus, Web of Science, Google Scholar, the Cochrane Library, ClinicalTrials.gov databases was conducted, including studies from 1996 to October 2024. Additionally, the citations from studies and reviews were also examined. Given the chronic nature of these pathologies, an emphasis was placed on studies with longer duration of follow-up. Inclusion criteria were (1) allogeneic OSST studies that included KLAL, lr-CLAL, allo-CLET, or allo-SLET surgeries with (2) at least 10 eyes with a (3) minimum mean/median follow-up of 24 months. Additionally, a success rate for the studied cohort had to be noted in the study or be calculated by the provided data. Exclusion criteria included (1) studies with mixed cohorts (multiple types of OSST surgeries) where the data was not available to determine the success of a particular type of OSST, (2) studies with autologous OSST surgeries, (3) studies with combined allogeneic OSST surgeries (e.g. combined lr-CLAL/KLAL), and (4) studies that evaluated only successful surgeries (for specific analysis). If data for separate procedures was provided in large series containing multiple types of surgeries, the data could be parsed out to include part of the cohort for a particular procedure if it met inclusion criteria (e.g. if a study had data listed for some eyes undergoing KLAL and other eyes undergoing lr-CLAL, the data was analyzed for all the KLAL eyes and included as a KLAL cohort while the same was done for the lr-CLAL eyes). When multiple articles were written by the same group of authors or institution, the review periods was assessed to ensure no overlap was included. The study with the most comprehensive data (ie. listed success rates, the largest series of eyes) was included to circumvent undue bias from any single institution. If a group published a separate type of surgery that clearly did not include eyes from another study (e.g. KLAL alone and combined modified KLAL with keratoplasty), these studies were included. Intermediate-term duration included mean follow-up from 2–5 years, and long-term duration included mean follow-up ≥ 5 years. Studies were assessed for bias via the Joanna Briggs Institute (JBI) critical appraisal tool (17). **Table 2** lists the questions in the JBI tool used to assess case series, and **Table 3** demonstrates the assessment of all included studies.

**Table 2:** Bias risk assessment questions from Joanna Briggs institute (JBI) critical appraisal tool for case series.

Were there clear criteria for inclusion in the case series? Was the condition measured in a standard, reliable way for all participants included in the case series? (ie. clinical assessment, impression cytology, in vivo confocal microscopy). Were valid methods used for identification of the condition for all participants included in the case series? (ie. clinical assessment, impression cytology, in vivo confocal microscopy). Did the case series have consecutive inclusion of participants? Did the case series have complete inclusion of participants? Was there clear reporting of the demographics of the participants in the study? Was there clear reporting of clinical information of the participants? Were the outcomes or follow-up results of cases clearly reported? Was there clear reporting of the presenting sites’/clinics’ demographic information? Was statistical analysis appropriate?

**Table 3:** Bias assessment of included studies by Joanna Briggs institute critical appraisal tool.

Study (Year)	Q1	Q2	Q3	Q4	Q5	Q6	Q7	Q8	Q9	Q10
Tsubota et al. (1999) (18)	Y	Y, C	Y, C	U	U	Y	Y	Y	N	Y
Ilari et al. (2002) (19)	Y	Y, C	Y, C	U	U	Y	Y	Y	N	n/a
Solomon et al. (2002) (20)	Y	Y, C/I	Y, C/I	Y	U	Y	Y	Y	N	Y
Holland et al. (2003) (21)	Y	Y, C/H	Y, C/H	Y	Y	U	Y	Y	N	Y
Maruyama-Hosoi et al. (2006) (22)	Y	Y, C/I	Y, C/I	Y	U	Y	Y	Y	N	Y
Shi et al. (2008) (23)	Y	Y, C/I	Y, C/I	Y	U	U	Y	Y	N	Y
Wylegala et al. (2008) (24)	N	Y, C/I	Y, C/I	Y	U	Y	U	N	N	n/a
Liang et al. (2009) (25)	U	Y, C/I	Y, C/I	Y	U	Y	Y	Y	N	U
Han et al. (2011) (26)	U	Y, C/I	Y, C/I	U	U	Y	Y	Y	N	Y
Javadi et al. (2011) (27)	Y	Y, C/I	Y, C/I	U	U	Y	Y	Y	Y	Y
Baradaran-Rafii et al. (2013) (28)	Y	Y, C/I	Y, C/I	Y	U	Y	Y	Y	N	n/a
Krysik et al. (2020) (29)	U	Y, C	Y, C	U	U	Y	Y	Y	N	Y
Cheung et al. (2020) (30)	Y	Y, C	Y, C	U	U	Y	U	Y	Y	Y
Li et al. (2022) (31)	Y	U	U	U	U	Y	Y	Y	N	Y
Karimian et al. (2023) (32)	Y	Y, C/I	Y, C/I	U	U	Y	Y	Y	Y	Y
Tran et al. (2024) (33)	Y	U	U	Y	Y	Y	Y	Y	Y	Y
Peng et al. (2024) (34)	Y	U	U	Y	U	Y	Y	Y	Y	Y
Daya et al. (2001) (35)	Y	U	U	U	U	Y	Y	Y	N	n/a
Samson et al. (2002) (36)	Y	Y, C	Y, C	U	U	Y	Y	Y	N	n/a
Santos et al. (2005) (37)	Y	Y, C	Y, C	U	U	Y	Y	Y	Y	Y
Scocco et al. (2008) (38)	Y	Y, C	Y, C	U	U	Y	Y	Y	N	Y
Javadi & Baradaran-Rafii (2009) (39)	Y	Y, C/I	Y, C/I	Y	U	Y	Y	Y	N	n/a
Moreira et al. (2015) (40)	Y	U	U	Y	Y	Y	Y	Y	Y	Y
El-Hofi et al. (2019) (41)	Y	U	U	U	U	Y	Y	Y	N	Y
Ozer (2020) (42)	Y	Y, C/I	Y, C/I	U	U	Y	Y	Y	N	Y
Daya et al. (2005) (43)	Y	Y, C	Y, C	U	U	Y	Y	Y	Y	n/a
Shimazaki et al. (2007) (44)	Y	Y, C/I	Y, C/I	U	U	Y	Y	Y	N	Y
Pauklin et al. (2010) (45)	Y	Y, C/I	Y, C/I	Y	U	Y	Y	Y	N	Y
Basu et al. (2012) (46)	Y	Y, C	Y, C	U	U	Y	Y	Y	N	Y
Shortt et al. (2008, 2014) (47, 48)	Y	Y, C/I/V	Y, C/I/V	U	U	N	Y	Y	N	n/a
Shanbhag et al. (2019) (49)	Y	U	U	U	U	N	Y	Y	N	U
Prabhasawat et al. (2021) (50)	Y	Y, C/I/V	Y, C/I/V	U	U	Y	Y	Y	N	Y
Riedl et al (2024) (51)	Y	U	U	U	U	Y	Y	Y	N	Y

Q, question; Y, yes; N, no; U, unclear; n/a, nonapplicable; C, clinical examination; H, histology; I, impression cytology; V, in vivo confocal microscopy.

### Outcome measures

The main outcome measure was success of the OSST surgery in preventing recurrence of LSCD. Following an OSST surgery, OSST graft failure was defined as recurrent diffuse late staining, conjunctivalization, CNV, and/or PEDs. OSST graft success or survival was noted in the setting of no signs of graft failure (late staining, conjunctivalization, CNV, and/or PEDs).

Visual outcomes considered were (1) improvement in best corrected visual acuity (BCVA), defined as an improvement in acuity by ≥ 2 lines, or (2) the percentage of eyes with BCVA ≥ 20/200 (ambulatory vision). In studies where these outcome measures were not available, we considered the mean preoperative and postoperative BCVA.

Additional information collected or calculated when assessing studies included mean/median follow-up, LSCD etiology, use of amniotic membrane (AM) during OSST, simultaneous keratoplasty at the time of OSST, adverse events, topical and systemic immunosuppression, and whether blood type (ABO) and human leukocyte antigen (HLA) matching were performed for living-related donors/recipient pairs. ABO compatibility was based on the major blood type antigens with type A able to receive type A or O; type B able to receive type B or O; type AB able to receive any ABO type; and type O only able to receive from type O. HLA compatibility was based on the similarity between the class I and II HLA types of two individuals.

Evaluated adverse events included rates of OSST rejection (the most common cause of allograft failure), infectious keratitis, and glaucoma. Clinical characteristics used to identify an episode of acute rejection included pain, reduced vision, or photophobia in addition to one or more of the following: edema and neovascularization of OSST segments, intense sectoral or 360-degrees of limbal injection, and an epithelial rejection line accompanied by conjunctival injection (28, 52). Chronic rejection was defined by low-grade inflammation in the setting of no to mild symptoms, with progressive corneal conjunctivalization, vascularization, loss of epithelial integrity, and/or graft thinning often resulting in gradual surface failure. Late-onset allograft failure was defined as partial or total failure in the absence of any other cause (infection, significant exposure, etc), attributed to asymptomatic chronic rejection or exhaustion.

## Results

### Allogeneic outcomes

Results of the literature review are listed in **Table 4**. There were 35 studies (n=1,268 eyes) that were included for this review: 17 KLAL (n=865), 10 lr-CLAL (n=246), 5 allo-CLET (n=86), and 3 allo-SLET (n=71 eyes) studies. KLAL and lr-CLAL were the two main allogeneic OSST techniques for bilateral LSCD. Combined intermediate-term and long-term success rates (**Tables 5**, **6**) for KLAL and lr-CLAL are 71% (603/847, ranged from 30-87% (18–34, 53, 54) and 66% (146/220, range 13-83%) (24, 30, 35–42), respectively. Improvement in BCVA by ≥ 2 lines ranged from 35-92% and 31-81% for KLAL and lr-CLAL, respectively. In a long-term comparison study between these two techniques, lr-CLAL demonstrated lower rejection rates, improved graft survival, and better BCVA compared with KLAL (30). Outcomes in this large retrospective comparison of lr-CLAL (63 eyes) and KLAL (224 eyes), with mean follow-up of 7.2 years demonstrated that 82.5% of lr-CLAL eyes maintained a stable ocular surface compared with 64.7% of KLAL eyes (30). More importantly, only 6.3% of lr-CLAL eyes demonstrated a failed ocular surface, compared with 15.6% of KLAL eyes, at last follow-up. A smaller proportion of lr-CLAL eyes (30.2% compared with 43.3%) developed an episode of acute rejection, and a greater proportion of these episodes resolved with treatment in the lr-CLAL group (79.0% compared with 53.6%). These lower rejection rate results were likely secondary to the transplantation of HLA- and ABO-matched tissue (30, 55).

**Table 4:** Results of literature review by surgery type.

KLAL studies
Total mentioned: 135 Series or case report: 39 Number of studies that met the criteria: 17 Excluded studies: 22
lr-CLAL studies
Total mentioned: 53 Series or case reports: 22 Number of studies that met the criteria: 10 Excluded studies: 12
Allo-CLET studies
Total mentioned: 49 Series or case report: 16 Number of studies that met the criteria: 5 Excluded studies: 11
Allo-SLET studies
Total mentioned: 30 Series or case reports: 13 Number of studies that met the criteria: 3 Excluded studies: 10

**Table 5:** Keratolimbal allograft outcomes for case series with ≥ 10 eyes and minimum follow-up of 24 months.

Study	Eyes	Mean follow up (Range; Months)	Etiology	Success rate (stable ocular surface)	2-line visual improvement* or ≥ 20/200†	AM	Simultaneous keratoplasty	Adverse events	Topical immunosuppression	Systemic immunosuppression
Tsubota et al. (1999) (18)	43	38 (12-75)	SJS, MMP, CTI	51% (22/43)	60% (26/43)*	Yes	PK (n=28)	PEDs 60%, OHT 37%	CsA, corticosteroid	CsA, corticosteroid
Ilari et al. (2002) (19)	23	60 (15-96)	SJS, CTI, MMP, atopy, other	30% (7/23)	43% (10/23)*	Yes (n=5)	PK (n=7), LK (n=1)	Rejection 39%, PEDs/keratolysis 13%, OHT 26%, MK 13%	CsA, corticosteroid	CsA, corticosteroid
Solomon et al. (2002) (20)	39	34 (12-118)	SJS, CTI, MMP, atopy, CL, other	47% (18/39) at 3 yrs, 24% (9/39) at 5 yrs	54% at 3 yrs, 45% at 5 yrs†	Yes	PK (n=23)	Rejection 14%, PEDs 36%, OHT 26%, MK 8%, CME 3%	Corticosteroid	CsA
Holland et al. (2003) (21)	31	36 (12-117)	Anirdia	74% (23/31)	87% (27/31)*	No	0	Rejection 30%, tube placement 32%	CsA, corticosteroid	CsA, azathioprine, corticosteroid
Maruyama-Hosoi et al. (2006) (22)	85	47 (NR)	SJS, MMP, CTI, other	66% (56/85)	NR	Yes	PK (n=7), LK (n=2)	Limbal graft changes (defect, edema, engorgement) 13%	CsA, corticosteroid	CsA, corticosteroid
Shi et al. (2008) (23)	39	32 (24-48)	CTI	41% (16/39)	28% (11/39) †	Yes	PK (n=23)	Rejection 49%, PED 21%, hyphema 28%, hypotony 18%	CsA, corticosteroid	CsA, corticosteroid
Wylegala et al. (2008) (24)	43	31 (6-72)	CTI, MMP, SJS, postinflammatory	46% (32/69)	53% (23/43) at 6 mo, 35% (15/43) at 12 mo*	NR	0	Chronic rejection		Systemic immunosuppression
Liang et al. (2009) (25)	12	61 (36-91)	CTI, SJS, idiopathic	83% (10/12)	92% (11/12)*	Yes	0	Rejection 17%, PED 75%, HTN 8%, GI 8%	Corticosteroid	Tacrolimus, MMF, corticosteroid
Han et al. (2011) (26)	24	47 (17-114)	SJS, CTI, other	33% (8/24)	45% (10/24)*	Yes, (n=15)	PK (45%)	Rejection 42%, PED 33%, keratolysis 8%, OHT 38%, MK 21%	Corticosteroid, CsA (if oral CsA stopped)	CsA, corticosteroid, MMF (if CsA not tolerated or acute rejection)
Javadi et al. (2011) (27)	40	66 (30-102)	CTI	80% (32/40)	NR	No	PK, LK, numbers unspecified	Rejection 10%, PED 3%, keratolysis, endophthalmitis 3%, OHT	Corticosteroid	CsA, MMF, corticosteroid
Baradaran-Rafii et al. (2013) (28)	45	26 (6-48)	CTI, SJS	73% (33/45)	93% (42/45)*	Yes	0	Rejection 18%, primary failure 11%, OHT 17%, MK 9%	Corticosteroid	Tacrolimus, MMF, corticosteroid
Borderie et al. (2019) (53)	8	130 (25-223)	CTI, SJS, MMP, severe MK	50% (4/8) at 3 yrs, 33% (3/8) at 5 yrs	13% (1/8)*	No	0	PED 63%, perforation 50%, MK 38%, RD 13%	Corticosteroid, CsA	CsA, corticosteroid, or chlorambucil
Ozer et al. (2020) (42)	9	95 (25-138)	CTI	67% at 1 yr, 53% at 1.5 yrs	67% (12/18 KLAL and lr-CLAL)*	Yes (n=1)	PK, (67%)	Rejection 56%, glaucoma, MK, renal toxicity 11%	Corticosteroid	CsA
Krysik et al. (2020) (29)	43	24 (1-60)	CTI, SJS, other	60% (26/43)	53% (23/43), ≥1 line improvment	No	0	Rejection 2%, PED 63%, OHT/glaucoma	Corticosteroid	CsA, azathioprine, MMF, corticosteroid
Cheung et al. (2020) (30)	224	95 (12-180)	Aniridia, CTI, SJS, CL	65% (145/224)	70% (156/224*	No	0	Rejection 43%	Corticosteroid, CsA or lifitegrast	Tacrolimus, MMF, corticosteroid
Li et al. (2022) (31)	24	47 (18-158)	CTI, other	71% (35/49 partial and stable surface)	69.4% 34/49*	No	LK (n=24)	Rejection 38% MK 21% keratolysis 13%, glaucoma 17%	Corticosteroid, tacrolimus	Corticosteroid
Karimian et al. (2023) (32)	108	82 (24-98)	CTI	70% (76/108)	NR	No	LK (n=40)	MK 6%	Corticosteroid	CsA (or tacroliumus), MMF, corticosteroid
Karimian et al. (2023) (54)	9	78 (60-96)	CTI	89% (8/9)	70% (7/9)*	No	PK (n=9), all with en bloc KLAL-PK	PED 33%	Corticosteroid	Tacroliumus, MMF, corticosteroid
Tran et al. (2024) (33)	22	34 (9-95)	CTI, aniridia, MMP, iatrogenic, other	68% (15/22)	82% (18/22)*	Yes	PK (n=12), LK (n=2)	PED 26%, keratolysis 7%, OHT/glaucoma 30%, MK 11%, UTI	Corticosteroid ± tacrolimus	Tacroliumus (only n=18), MMF, corticosteroid
Peng et al. (2024) (34)	20	68 (17-99)	SJS	87% (NR)	75% (15/20)*	Yes	PK (n=3), LK (n=10)	Rejection 5% PED 20%, keratolysis 10%, OHT/Glaucoma 45%	Corticosteroid, tacrolimus	Corticosteroid

2-line visual improvement* or ≥ 20/200†. AM, amniotic membrane; SJS, Stevens Johnson syndrome; MMP, mucous membrane pemphigoid; CTI, chemical thermal injury; CL, contact lens; PK, penetrating keratoplasty; LK, lamellar keratoplasty; PED, persistent epithelial defect; OHT, ocular hypertension; CsA, cyclosporine; MMF, mycophenolate mofetil; MK, microbial keratitis; UTI, urinary tract infection; HTN, hypertension; GI, gastrointestinal; NR, not recorded.

**Table 6:** Living-related conjunctival limbal outcomes for case series with ≥ 10 eyes and minimum follow-up of 24 months.

Study	Eyes	Mean follow up (Range; Months)	Etiology	Success rate (stable ocular surface)	2-line visual improvement* or ≥ 20/200†	AM	Simultaneous keratoplasty	Adverse events	ABO/HLA matched	Topical immunosuppression	Systemic immunosuppression
Daya et al. (2001) (35)	10	26 (17-43)	SJS, ectodermal dysplasia, CTI, MMP, others	80% (8/10)	70% (7/10)*	No	0	Rejection 20%, PED/perforation 30%, MK 10%	ABO, HLA	Corticosteroid, CsA (n=1)	CsA, corticosteroid
Samson et al. (2002) (36)	11	35 (29-51)	SJS, atopic, HSV, Moorens	55% (6/11)	55% (6/11)	Yes (n=5)	0	Rejection 18%, PED 6%, MK 27%	HLA	Corticosteroid	CsA (n=3), azathioprine (n=2), CsA + azathioprine (n=2), methotrexate (n=1)
Santos et al. (2005) (37)	23	33 (NR, ±12)	SJS, CTI	13% (3/23)	NR	Yes	PK (NR)	MK 17% rejection 13%, graft necrosis 9%	HLA	Corticosteroid	CsA (n=17), corticosteroid
Scocco et al. (2008) (38)	39	49 (18-121)	SJS, CTI, iatrogenic, others	79% (31/39), 13 eyes (33.3%) required 2^nd^ lr-CLAL	31% (12/39)*	Yes (n=7)	PK (n=1)	Rejection 18%, endophthalmitis 3%	HLA	NR	None
Wylegala et al. (2008) (24)	26	31 (6-72)	SJS, MMP, CTI, others	46% (32/69)‡	54% (14/26)*	NR	NR	Rejection 27%, PED 35%	HLA	NR	Systemic immunosuppression
Javadi & Baradaran-Rafii (2009) (39)	25	37 (12-78)	CTI	80% (20/25)	52% (13/25)*	No	PK (n=5), LK (n=2)	Acute Rejection 40%, chronic rejection 32%, MK 4%, OHT 12%	None, 1^st^ degree siblings	Corticosteroid	CsA, corticosteroid
Moreira et al. (2015) (40)	13	35 (6-NR)	SJS, aniridia, iatrogenic	23% (3/13)	NR	Yes (n=9)	0	PED 39%, keratolysis 23%, graft necrosis 15%, descemetocele 15%, MK 8%, perforation 8%	HLA	Corticosteroid	Systemic immunosuppression
El-Hofi et al. (2019) (41)	20	29 (18-42)	CTI	75% (15/20)	80% (16/20)*	Prior (n=20)	0	Acute rejection 15%, OHT/glaucoma 35%, graft failure 25%	HLA	Corticosteroid	CsA, corticosteroid
Cheung et al. (2020) (30)	63	60 (12-192)	Aniridia, CTI, SJS, contact lens, others	83% (52/63)	81.1% (51/63)*	No	0	Acute rejection 30%	ABO, HLA	Corticoesteroid, CsA or lifitegrast	Tacrolimus, MMF, corticosteroid
Ozer (2020) (42)	16	92 (25-151)	CTI	65% at 1 yr, 54% at 2 yrs, 37% at 3 yrs	67% (12/18 KLAL and lr-CLAL)*	Yes (n=5)	No	Rejection 94%, glaucoma, MK, renal toxicity 6%	HLA	Corticosteroid	CsA

‡KLAL, lr-CLAL results reported together. *2-line visual improvement; †≥20/200. AM, amniotic membrane; SJS, Stevens Johnson syndrome; MMP, mucous membrane pemphigoid; CTI, chemical thermal injury; CL, contact lens; PK, penetrating keratoplasty; LK, lamellar keratoplasty; PED, persistent epithelial defect; OHT, ocular hypertension; CsA, cyclosporine; MMF, mycophenolate mofetil; MK, microbial keratitis; NR, not recorded.

Due to these advantages, we consider lr-CLAL our first option for patients with total LSCD and a compatible living related donor. For allogeneic OSST, systemic immunosuppression is necessary, and employing a triple therapy regimen demonstrated higher long-term graft survival/ocular surface stability results, ranging from 65-83% for KLAL (25, 27, 30, 32) and 83% for lr-CLAL (30) compared to those long-term studies utilizing only 1 or 2 immunosuppression agents (mean 47%, range 24-87%) (19, 20, 33). Focusing on the importance of immunosuppression, Javadi et al. attributed their finding of a 39.1% lr-CLAL group survival compared to an 80.7% KLAL group survival to the fact that the KLAL group used cyclosporine A, mycophenolate mofetil, and corticosteroids for immunosuppression compared to only cyclosporine A and corticosteroids in the lr-CLAL group (27). **Table 7**, which totals the cumulative intermediate- and long-term stability results from the previous tables, also highlights the greater long-term success rate with triple agent immunosuppression compared to single or dual agent immunosuppression for allografts. Studies have consistently shown inflammatory and cicatrizing conditions such as Stevens Johnson syndrome, mucous membrane pemphigoid, and severe chemical injuries to have the worst outcomes following OSST (16, 20). Additional risk factors for failure (particularly for KLAL) include keratinization, decreased mucin and aqueous tear deficiency, symblepharon, chronic conjunctival inflammation, prior rejection, lid abnormalities, and preoperative increased intraocular pressure (56).

**Table 7:** Cumulative intermediate- and long-term stability results for allogeneic OSST techniques.

Technique	Intermediate-term stability 2-4 years (n)	Long-term stability ≥5 years (n)
OSST Allograft (Single/dual systemic immunosuppression regimen)	KLAL: 54% (191/356) Lr-CLAL: 56% (118/210)	KLAL: 40% (36/90)
OSST Allograft (Triple systemic immunosuppression regimen)	KLAL: 69% (97/141) Lr-CLAL: No data	KLAL: 69% (271/393) Lr-CLAL: 83% (52/63)
CLET Allograft	52% (63/122)	0% (0/7)
SLET Allograft	81% (38/47)	No Data

Allogeneic-CLET (allo-CLET) is a procedure that involves *ex vivo* expansion of 1–2 mm biopsies from a donor corneoscleral ring to produce a sheet of stem cells (5). It has been speculated that there might be a reduced risk of allograft rejection when using *ex vivo* cultivated cells, which could be explained by the absence of antigen-presenting Langerhans cells (57). The allo-CLET intermediate-term success rates (stable surface) ranged from 25-70% (43–48, 57). No long-term results have been published in sizeable cohorts. Visual results included a range of 40-64% demonstrating a 2-line visual improvement (see **Table 8**). Systemic immunosuppression regimens varied but typically utilized 1–2 agents (including corticosteroids). The absence of an established limbal niche may play a role in the less successful long-term results.

**Table 8:** Allogeneic CLET outcomes for case series with ≥ 10 eyes and minimum follow-up of 24 months.

Study	Eyes	Mean follow up (Range; Months)	Etiology	Success rate (stable ocular surface)	2-line visual improvement* or ≥ 20/200†	AM	Simultaneous keratoplasty	Adverse events	ABO/HLA matched	Topical immunosuppression	Systemic immunosuppression
Daya et al. (2005) (43)	10	28 (12–50)	Ectodermal dysplasia, SJS, CTI, others	70% (7/10)	40% (4/10)*	Yes (100%)	0	MK 10%, PED 30%	No; first degree relative selected for living related donors	Corticosteroid	CsA, corticosteroid
Shimazaki et al. (2007) (44)	20	32 (7–92)‡	SJS, MMP, CTI, others	50% (10/20)	48% (13/27) * ‡	Yes (100%)	0	PED/ulcer 20%, MK 15% corneal perforation 20% ‡	No; first degree relative selected for living related donors	Corticosteroid, CsA	CsA, corticosteroid
Pauklin et al. (2010) (45)	14	29 (NR)	CTI, aniridia, iatrogenic, others	50% (7/14)	64% (9/14)*	Yes (100%)	0	Perforation 14%, rejection 7%, bleeding beneath AM 7%	NR	Corticosteroid	CsA or MMF
Basu et al. (2012) (46)	28	58 (12–114)	CTI, allergic, CL, SJS, others	64% (18/28) at 3 yrs	43% (12/28) †	Yes (100%)	0	PED with melting 25%, rejection 7%, OHT 7%	No; first degree relative selected for living related donors	Corticosteroid	CsA, corticosteroid
Prabhasawat et al. (2012) (50)	7	25 (11-47)	SJS, CTI, iatrogenic, allergic	86% (6/7)	86% (6/7)*	Yes (100%)	0	MK 14%	No, all deceased donors	Corticosteroid	CsA
Shortt et al. (2008, 2014) (47, 48)	14	36 (NR)	SJS, aniridia	25% (NR)	57% (8/14)*	Yes (100%)	0	NR	No, all deceased donors	Corticosteroid	CsA or MMF, corticosteroid

‡autologous and allogeneic results reported together. ‡KLAL, lr-CLAL results reported together. 2-line visual improvement* or ≥ 20/200†. AM, amniotic membrane; SJS, Stevens Johnson syndrome; MMP, mucous membrane pemphigoid; CTI, chemical thermal injury; CL, contact lens; PK, penetrating keratoplasty; LK, lamellar keratoplasty; PED, persistent epithelial defect; OHT, ocular hypertension; CsA, cyclosporine; MMF, mycophenolate mofetil; MK, microbial keratitis; NR, not recorded.

SLET is a newer technique of *in vivo* limbal stem cell expansion. Given the relative newness of this technique, there are only a few case series of allogeneic-SLET (allo-SLET) with intermediate-term follow-up in the literature and no studies with long-term data (49–51). While success rates are encouraging for these early studies and range from 54-83% (**Table 9**), future long-term studies will be necessary to determine the longevity of allo-SLET. Systemic immunosuppression regimens vary but typically utilize 1–2 agents (including corticosteroids). It is important to point out that SLET (like CLET) only provides LSCs and does not provide conjunctiva or conjunctival stem cells. Therefore, the cases reported with SLET and CLET often have much milder ocular surface disease without significant conjunctival involvement than cases managed with lr-CLAL or KLAL. It is also crucial to remember that conjunctival severity is the most important predictor of OSST outcomes.

**Table 9:** Allogeneic SLET outcomes for case series with ≥ 10 eyes and minimum follow-up of 24 months.

Study	Eyes	Mean follow up (Range; Months)	Etiology	Success rate (stable ocular surface)	Mean pre- and post-operative BCVA*	AM	Simultaneous keratoplasty	Adverse events	ABO/HLA matched	Topical immunosuppression	Systemic immunosuppression
Shanbhag et al. (2019) (49)	30	28 (13-66)	SJS, MMP, CTI, allergic	83% (25/30)	HM → 20/80	Yes (100%)	0	Hemorrhage under AM 30%, rejection 7%	No; first degree relative selected for living related donors	Corticosteroid	CsA, pulsed corticosteroid
Prabhasawat et al. (2021) (50)	17	29 (17-37)	CTI, SJS, MGD, aniridia, allergic, iatrogenic	76% (13/17)	CF →20/160	Yes (100%)	0	Limbal explant loss, acute rejection 18%, glaucoma, failure, shingles 6%, CMV	ABO; first degree relative selected for living related donors	Corticosteroid	MMF +/- CsA, corticosteroid

*No available data for either number of eyes with 2-line visual improvement or ≥ 20/200. AM, amniotic membrane; SJS, Stevens Johnson syndrome; MMP, mucous membrane pemphigoid; CTI, chemical thermal injury; CF, counting fingers; HM, hand motions; CMV, cytomegalovirus; CsA, cyclosporine; MMF, mycophenolate mofetil.

### Rejection outcomes

The major complication of allograft procedures is the high risk of immune rejection (16, 52). Rejection rates for traditional allograft OSST surgeries (KLAL, lr-CLAL, combined procedures) range from 2 -94% (19–21, 23–31, 35–39, 41, 42, 53). Most studies did not differentiate between acute and chronic rejection. For allo-CLET and allo-SLET, there are decreased rates of reported rejection despite comparable (for intermediate-term) survival rates compared to KLAL/lr-CLAL. Rejection may be more difficult to detect for these surgeries, or failure could be mainly related to other factors (e.g. cell exhaustion). Ang et al. found rejection to be a risk factor for a failed surface (only 36.6% in the rejection group achieved a stable ocular surface compared with 71.9% in the nonrejection) despite increased immunosuppression and repeat OSST (52). Risk factors for rejection were younger age, KLAL alone compared to lr-CLAL (or combination procedures), and systemic immunosuppression nonadherence.

**Supplementary Tables 1**–**4** list the rejection presentations defined by each study and the typical treatment. As the transplanted tissue varies by type of OSST, the presentation of rejection also varies. In general, there is pain, conjunctival injection or subconjunctival hemorrhage accompanying focal edema/congestion of the transplanted tissues especially for KLAL and lr-CLAL. Signs of epithelial rejection (rejection line), corneal neovascularization, epithelial haze, epithelial irregularity or frank defect, and decreased vision could be seen with all types of OSST. OSST segment injection with accompanying subconjunctival hemorrhage and edema to the segments should be more concerning for rejection compared to other forms of injection. Similarly, sludging is a noninflammatory vascular phenomenon seen in KLAL (more common) and lr-CLAL characterized by dilated blood vessels localized to the graft (58). There is absence of inflammation, limbal injection, and corneal epithelial abnormalities. This does not represent rejection, but we have shown that these eyes may have a higher chance of rejection in the future. Common forms of rejection treatment consisted of systemic and topical corticosteroids (and even periocular administration) with many groups also augmenting systemic immunosuppression. Some of us have also added intravenous immunoglobulin (IVIG) to our rejection treatment protocol with success, especially in cases refractory to the above mentioned treatments (59, 60).

Matching blood type and HLA compatibility likely significantly reduces the risk of rejection in deceased donor ocular tissue. Mimouni et al. demonstrated that KLAL outcomes and rejection rate could be improved through compatibility matching and following an innovative immunosuppressive protocol, a modification on the Cincinnati protocol (3 immunosuppressive agents: mycophenolate, tacrolimus, and prednisone) with perioperative basiliximab and intravenous immunoglobulin (IVIG) as indicated by increased risk (61). In renal transplantation, basiliximab is an immunosuppressant monoclonal antibody used to prevent early acute transplant rejection by blocking the IL-2 receptor on activated T-lymphocytes. IVIG, a pooled product of blood serum from numerous human donors with the primary composition proteins being immunoglobulins and albumin, can block the binding of donor specific antibodies, help with the regulation of T and B lymphocytes, and decrease episodes of acute rejection. Unfortunately, one must have a high suspicion for rejection signs and symptoms as early or chronic/smoldering rejection may go unnoticed; thus, physicians should be aware to closely monitor patients and aggressively treat rejection cases with increased topical and systemic immunosuppression for better outcomes (16). Late failure (exhaustion of transplanted cells) can be seen with recurrence of the LSCD and late staining, but the eye is typically quiet compared to chronic rejection.

### Ocular hypertension/glaucoma

Tsai et al. found a high prevalence (65.7%) of ocular hypertension (OHT) and glaucoma in LSCD patients (63). To improve long-term ocular surface outcomes, an optimal intraocular pressure prior to OSST must be achieved. Early placement of tube shunts prior to an OSST may be helpful. The cause of OHT/glaucoma following OSST was often attributed to corticosteroid response although there were also underlying etiologies (e.g. aniridia, chemical injury) or anatomical changes (post-PK) that could also play a role (29, 33, 34, 45, 46). Rates of OHT or glaucoma following OSST range from 12-45% (18–21, 26, 28, 31, 33, 39, 41, 54), and there is a significant proportion requiring glaucoma drainage device (GDD) surgery (33-90%) (18, 20, 26). **Table 10** highlights the various glaucoma treatments that were used in the reviewed studies. It has been shown that ciliary sulcus tube placement helps preserve the health of a pre-existing OSST while minimizing the risk of tube-endothelium contact to maximize future keratoplasty success (64). While GDD surgery may offer more substantial IOP reduction, transscleral cyclophotocoagulation (CPC) can be a safe option for the management of secondary glaucoma after OSST (65). G-probe and/or micropulse CPC may have a lower rate of complications requiring incisional repair than GDD and should be considered particularly in eyes with severe conjunctival disease. It is important to discuss preoperatively with the glaucoma specialist regarding the location of the OSST surgery if transscleral CPC is necessary post-OSST to minimize damage to the transplanted stem cells. Micropulse CPC may have a better safety profile as it is placed 3 mm from the limbus compared to the G-probe CPC (1 mm from the limbus).

**Table 10:** Ocular hypertension and glaucoma treatments in OSST studies.

Study	OHT/Glaucoma treatment
Tsubota et al. (1999) (18)	Surgery (no details), Topical treatment
Ilari et al. (2002) (19)	Topical treatment
Solomon et al. (2002) (20)	GDD (Baerdvelt implant)
Holland et al. (2003) (21)	GDD
Liang et al. (2009) (25)	Topical treatment
Han et al. (2011) (26)	6/9 Topical treatment. 3/9 GDD or CPC.
Javadi et al. (2011) (27)	5/8 Topical treatment. 2/8 Trabeculectomy with MMC. 1/8 GDD.
Baradaran-Rafii et al. (2013) (28)	8/8 Topical treatment. 4/8 GDD. 1/8 CPC. 1/8 Cyclocryotherapy.
Krysik et al. (2020) (29)	25/25 Topical treatment. 3/25 Trabeculectomy. 2/25 Transscleral CPC. 3/25 Express shunt.
Li et al. (2022) (31)	No details
Tran et al. (2024) (33)	GDD
Peng et al. (2024) (34)	11/12 Topical treatment. 1/12 GDD.
Javadi & Baradaran-Rafii (2009) (39)	No details
El-Hofi et al. (2019) (41)	5/7 GDD (Ahmed valve). 2/7 Topical treatment.
Ozer et al. (2020) (42)	Topical treatment, GDD, CPC
Basu et al. (2012) (46)	Oral or topical treatment
Prabhasawat et al. (2021) (50)	Topical treatment

OHT, ocular hypertension; GDD, glaucoma drainage device; CPC, cyclophotocoagulation; MMC, mitomycin C.

### Infectious keratitis

Although there may be a decreased risk of microbial keratitis (MK) following OSST (compared to non-OSST LSCD eyes), there is a higher incidence seen in OSST eyes compared to PK eyes (without OSST) and the general population (62). Rates of MK following OSST range from 4-43% (19, 20, 26, 28, 31, 32, 35–37, 39, 40, 42–44, 49, 54), and severe MK can lead to OSST and/or corneal graft failure (20, 35–37, 54). The various types of MK encountered in the reviewed studies are listed in **Table 11**. In the largest study specifically reviewing MK incidence (19%, 52/278 eyes) and outcomes in OSST, Cheung et al. reported a high percentage of bacterial-related cases to be gram-positive species, especially Streptococcus and Staphylococcus species (66). While most cases were of bacterial etiology, there were also a significant number (nearly 1/3) of fungal cases, particularly in LSCD eyes with an underlying cicatrizing etiology. Cicatrizing etiologies also accounted for the highest rates of MK (72% of cases overall) and comprised 93% of eyes developing multiple episodes of MK. With early and aggressive management, most MK cases resolved with antimicrobial treatment; however, therapeutic keratoplasty was sometimes necessary, especially in fungal cases. In other studies, similar treatment was necessary for fungal infections including topical anti-fungals and at times keratoplasty (20, 36). Viral keratitis has also been noted postoperatively. HSV keratitis episodes were seen in 9% of eyes by Cheung et al (66), 11% (2/18) by Ozer et al (42), 4% (1/24) by Han et al (26), and 9% (4/45) by Baradaran-Rafii et al. (28) Prabhasawat et al. noted one case of cytomegalovirus and one case of zoster while Baradaran-Rafii et al. noted 2 cases of papillomavirus (28, 50).

**Table 11:** Etiologies of microbial keratitis in OSST studies.

Study	Infectious etiology
Ilari et al. (2002) (19)	1 case of Haemophilus influenzae. 2 cases of coagulase negative Staphylococci.
Solomon et al. (2002) (20)	2 cases of candida species. 1 case of coagulase negative Staphylococci.
Han et al. (2011) (26)	5 bacterial keratitis. 1 case of fungal keratitis. 1 case of HSV.
Baradaran-Rafii et al. (2013) (28)	4 cases of HSV. 2 cases of papillomavirus.
Li et al. (2022) (31)	No details
Karimian et al. (2023) (32)	No details
Tran et al. (2024) (33)	Fungal keratitis
Daya et al. (2001) (35)	Pseudomonas
Samson et al. (2002) (36)	Mycobacterium, fungus
Santos et al. (2005) (37)	1 case of Acanthamoeba mixed with Candida species and Enterobacter species. 1 case Candida species. 1 case of Staphylococcus epidermidis. 1 case of Pseudomonas Aeruginosa.
Javadi & Baradaran-Rafii (2009) (39)	No details
Moreira et al. (2015) (40)	No details
Ozer et al. (2020) (42)	1 bacterial case, 2 HSV cases
Daya et al. (2005) (43)	Bacterial keratitis
Shimazaki et al. (2007) (44)	No details
Prabhasawat et al. (2021) (50)	1 case of VZV 1 case of CMV

HSV, herpes simplex virus; VZV, varicella zoster virus; CMV, cytomegalovirus.

### Limitations

Limitations of this review include the inherent retrospective nature of included studies where the available presented data for each reviewed study confined our inclusion, analysis, and conclusions that could be made. Due to the relatively uncommon nature of severe LSCD needing OSST and most patients being treated at isolated centers, data on these procedures was published only in case series format with no published randomized clinical trial data to date. Our outcomes were limited to the most commonly considered outcomes for OSST studies where most studies diagnosed recurrence of LSCD clinically without adjunctive assessments such as impression cytology, anterior segment optical coherence tomography, or *in vivo* confocal microscopy. If impression cytology was used, many studies only used it for a couple to handful of eyes and not every eye in the case series. Early in the understanding of LSCD (about 30–40 years ago) impression cytology was needed to identify and diagnose LSCD; however, with increased experience, we would agree with other experienced groups that this is not necessary clinically to diagnose LSCD and gauge the success of OSST grafts. Certain details such as HLA matching and duration of systemic or topical therapy were difficult to compare as there was either a lack of details provided on the process or varying protocols utilized among the various studies. Additionally, there is inherent variability in the criteria for LSCD, ocular surface stability, and diagnosis of rejection among studies that may affect the actual comparison of success rates. Similarly, more aggressive systemic immunosuppression regimens may have been used at specialized centers or for more advanced cases. Lastly, this review focused on limbal based surgeries, so oral mucosal and mesenchymal stem cell (MSC) transplantation techniques were not included although these procedures could be used for bilateral stem cell disorders. At the time of the review, there were only intermediate-term studies available for cultivated oral mucosal epithelial transplantation (COMET, **Table 12**), and neither intermediate-term or long-term studies were available for MSC transplantation.

**Table 12:** Cultivated oral mucosal epithelial transplantation (COMET) outcomes for case series with ≥ 10 eyes and minimum follow-up of 24 months.

Study	Eyes	Mean follow up (Range; Months)	Success rate (stable ocular surface)	2-line visual improvement
Nakamura et al. (2011) (67)	19	55 (36-90)	Not recorded (NR)	42% (8/19)
Satake et al. (2011) (68)	40	26 (6-55)	58% (23/40)	NR
Prabhasawat et al. (2016) (69)	20	32 (8-50)	75% (15/20)	70% (14/20)
Baradaran-Rafii et al. (2017) (70)	14	28 (14-40)	93% (13/14)	NR

## Conclusion

Allogeneic OSST can successfully achieve long-term ocular surface stability. Success requires adequate systemic immunosuppression, appropriate monitoring for postoperative adverse events, and committed patient adherence. Lr-CLAL and KLAL are excellent allogeneic procedures that have been shown to have the best long-term OSST outcomes. Both lr-CLAL and KLAL can restore the ocular surface and benefit from 3-agent systemic immunosuppression to optimize long-term results. Besides the ability to treat conjunctival disease, lr-CLAL can be superior to KLAL due to lower rates of rejection leading to better ocular surface results, as seen in the largest comparative study to date; however, additional studies should confirm these findings.

Allo-CLET demonstrates fair intermediate-term results, comparable to KLAL and lr-CLAL without 3-agent systemic immunosuppression. Allo-CLET and allo-SLET currently lack long-term results in large cohorts. Managing adverse events such as rejection, glaucoma, and MK is important to improve long-term visual outcomes.

## Supplementary material

Supplementary Table 1 Rejection rates, characteristics, and treatment for keratolimbal allografts case series with ≥ 10 eyes and minimum follow-up of 24 months.

Supplementary Table 2 Living related conjunctival limbal rejection rates, characteristics, and treatments for case series with ≥ 10 eyes and minimum follow-up of 24 months.

Supplementary Table 3 Allogeneic CLET rejection rates, characteristics, and treatments for case series with ≥ 10 eyes and minimum follow-up of 24 months.

Supplementary Table 4 Allogeneic SLET rejection rates, characteristics, and treatments for case series with ≥ 10 eyes and minimum follow-up of 24 months.
