# Supplementary material for: Outcomes of allogeneic ocular surface stem cell transplantation
Source: Front Ophthalmol (Lausanne). 2026 Jun 11;6:1836045. doi: 10.3389/fopht.2026.1836045 (PMC13293912; doi:10.3389/fopht.2026.1836045)
Supplement: Supplementary Table 1 — Rejection rates, characteristics, and treatment for keratolimbal allografts case series with ≥ 10 eyes and minimum follow-up of 24 months. [file Table1.docx]

**Supplemental Table 1. Rejection Rates for Keratolimbal allografts for case series with ≥ 10 eyes and minimum follow-up of 24 months**

|  | Rejection Rate (eyes) | Signs of Rejection | Treatment |
| --- | --- | --- | --- |
| Tsubota et al. (1999)^19^ | 13/28 (46%) | Classic epithelial appearance of rejection, accompanied by stromal edema in the transplanted corneal button | Intravenous and topical corticosteroids |
| Ilari et al. (2002)^20^ | 9/23 (39%) | Pain, photophobia, sectorial conjunctival injection, and edema with local epitheliopathy leading to an epithelial defect | Oral CsA |
| Solomon et al. (2002)^21^ | Limbal allograft rejection: 3/21 (14%) | KLAL: congestion of the peri-limbal and limbal blood vessels and swelling of the tissue | Not defined |
| Holland et al. (2003)^22^ | (7/23) 30% | Not defined | Increasing oral prednisone and topical corticosteroids |
| Maruyama-Hosoi et al. (2006)^23^ | 16/121 (13%) | Epithelial defects, acute edema and vascular engorgement | Increasing frequency of topical steroids |
| Shi et al. (2008)^24^ | Limbal stem cell rejection in PK + KLAL: 15/23 (65%) | Limbal stem cell rejection: pain, photophobia and lacrimation, obvious sectoral or circular limbal congestion and oedema with subconjunctival hemorrhage, epithelial edema and epithelial defect or epithelial rejection lines | IV systemic cortisol (2 mg/kg )for 3 days then oral prednisone of 0.5 mg/kg daily for up to 1 month. Topical tobramycin and dexamethasone eye drops and CsA 0.5% eyedrops four times a day |
| Wylegala et al. (2008)^25^ | 7/43 (16%) | Not defined | Not defined |
| Liang et al. (2009)^26^ | KLAL: 2/12 (17%) | Not defined | Daily doses of 1.1 mg of Mycophenolate Mofetil and 1.8 mg of tacrolimus |
| Han et al. (2011)^27^ | 10/24 (42%) | Swollen or opaque limbal grafts combined with limbal vascular congestion | Oral CSA dose was increased, oral prednisolone was restarted, and prednisolone acetate eye drops were instilled more frequently. Oral mycophenolate mofetil 1.0 gram twice a day was also started |
| Javadi et al. (2011)^28^ | Ir-CLAL: 10/32 (31%)  KLAL: 4/40 | Acute Stem Cell Rejection: Limbal and perilimbal vascular engorgement and conjunctival chemosis | Increasing dosage and/or frequency of topical and systemic corticosteroids |
| Baradaran-Rafii et al. (2013)^29^ | 8/45 (17%) | Regional or 360-degree KLAL swelling and hyperemia accompanied by moderate to severe conjunctival injection, pain, and sometimes photophobia | Increasing dosage and/or frequency of topical and systemic steroids and systemic immunosuppression |
| Krysik et al. (2020)^31^ | 4/43 (9%) | Epithelial rejection | Intensive topical and systemic corticosteroids |
| Cheung et al. (2020)^32^ | 43% (97/224) | Intense sectoral or 360 degrees of limbal injection, and an epithelial rejection line accompanied by conjunctival injection | Not defined |
| Li et al. (2022)^33^ | 4/24 (16%) | Not defined | Short-term courses of high-dose corticosteroids and tacrolimus eye drops |
| Karimian et al. (2023)^34^ | 0/9 (%) | Not defined | Not defined |
| Tran et al. (2024)^36^ | Acute Rejection: (0/27) 0%  Chronic Rejection: (7/27) 26% | Acute Rejection: pain, photophobia, sectoral conjunctival injection, limbal edema and/or infiltration, rapid-onset or persistence of large epithelial defects, or epithelial rejection lines  Chronic rejection: progressive corneal conjunctivalization, neovascularization, progressive whorl-like keratopathy, or low-grade limbal edema or injection, | Increase in topical and/or systemic immunosuppression |
| Peng et al. (2024)^37^ | 1/24 (4%) | Not defined | Not defined |
