# Supplementary material for: Outcomes of allogeneic ocular surface stem cell transplantation
Source: Front Ophthalmol (Lausanne). 2026 Jun 11;6:1836045. doi: 10.3389/fopht.2026.1836045 (PMC13293912; doi:10.3389/fopht.2026.1836045)
Supplement: Supplementary Table 2 — Living related conjunctival limbal rejection rates, characteristics, and treatments for case series with ≥ 10 eyes and minimum follow-up of 24 months. [file Table2.docx]

**Supplemental Table 2. Living-related conjunctival limbal rejection rates for case series with ≥ 10 eyes and minimum follow-up of 24 months**

|  | Rejection Rate (eyes) | Signs of Rejection | Treatment |
| --- | --- | --- | --- |
| Daya et al. (2001)^36^ | 4/10 (40%) | Pain, sectoral conjunctival injection, edema, and local epithelial abnormality leading to an epithelial defect | High-dose systemic corticosteroids, increased dose of oral cyclosporine to 5 mg/kg/day, and intensive topical steroid |
| Samson et al. (2002)^37^ | 2/11 (18%) | Focal graft edema and neovascularization growing over the graft and onto the cornea | Increased dosage of topical steroids |
| Santos et al. (2005)^38^ | 3/33 (9%) | Not defined | Increase in oral cyclosporine and topical corticosteroid |
| Scocco et al. (2008)^39^ | 7/39 (17.9%) | Ischemic graft or intense vessel dilation adjacent to the graft was present | Corticosteroid drops |
| Wylegala et al. (2008)^25^ | 7/26 (27%) | Not defined | Not defined |
| Javadi & Baradaran-Rafii (2009)^40^ | 10/25 (40%) | Limbal and perilimbal vascular engorgement and conjunctival chemosis in the transplant | Increased dosage of topical and systemic corticosteroids that was tapered with elimination of vascular engorgement and conjunctival chemosis |
| Moreira et al. (2015)^41^ | N/A | Not defined | Not defined |
| El-Hofi et al. (2019)^42^ | 3/20 (15%) | Pain, decreased vision, photophobia, intense ciliary injection, and edema of the graft. | More frequent topical steroid eyedrops (1% Prednisolone acetate), subconjunctival injection of triamcinolone, oral corticosteroids with tapering over several months, and increasing the dose of the oral immunosuppressive agents |
| Cheung et al. (2020)^31^ | Ir-CLAL: 30% (19/63) | Pain, decreased vision, or photophobia in addition to one or more of the following: edema and neovascularization of OSST segments | Not defined |
| Ozer (2020)^43^ | Ir-CLAL: 15/21 (71%)  KLAL: 5/9 (55%) | Graft edema along with failure to maintain normal corneal epithelium and conjunctivalization recurrence | Systemic corticosteroids were started, and if the systemic immunosuppressive dosage was being tapered during a rejection incident, the dosage was increased to the initial levels and then continued until the repetition of allograft transplantation and again gradually tapered at least 18 months after the surgical procedure. |
