# Supplementary material for: Outcomes of allogeneic ocular surface stem cell transplantation
Source: Front Ophthalmol (Lausanne). 2026 Jun 11;6:1836045. doi: 10.3389/fopht.2026.1836045 (PMC13293912; doi:10.3389/fopht.2026.1836045)
Supplement: Supplementary Table 3 — Allogeneic CLET rejection rates, characteristics, and treatments for case series with ≥ 10 eyes and minimum follow-up of 24 months. [file Table3.docx]

**Supplemental Table 3.** **Allogeneic CLET rejection and failure rates for case series with ≥ 10 eyes and minimum follow-up of 24 months**

|  | Rejection Rates | Signs of Rejection | Treatment |
| --- | --- | --- | --- |
| Daya et al. (2005)^47^ | Failure Rates:  3/10 (30%) | Signs of Failure: Development of a PED | Large Defects: PK, Lamellar keratoplasty, KLAL and AMT  Small Defects: bandage contact lens |
| Shimazaki et al. (2007)^48^ | Failure Rates:  (10/20) 50% | Signs of Failure: No stable epithelium with corneal phenotype on the central cornea or peripheral conjunctival invasion. | Not defined |
| Pauklin et al. (2010)^49^ | 7% (1/14) | Engorged limbal vessels, impairment of ocular surface integrity and an acute advancement of conjunctival tissue onto the reconstructed corneal surface | Local and systemic administration of corticosteroids |
| Basu et al. (2012)^50^ | 7% (2/28) | Epithelial rejection | Medically treated |
| Shortt et al. (2008, 2014)^51,52^ | Failure Rate: 75% (NR) | Signs of Failure: Presence of epithelial haze, superficial neovascularisation, epithelial irregularity, and epithelial defect. Each was correlated with visual acuity. | Not defined |
