# Supplementary material for: Outcomes of allogeneic ocular surface stem cell transplantation
Source: Front Ophthalmol (Lausanne). 2026 Jun 11;6:1836045. doi: 10.3389/fopht.2026.1836045 (PMC13293912; doi:10.3389/fopht.2026.1836045)
Supplement: Supplementary Table 4 — Allogeneic SLET rejection rates, characteristics, and treatments for case series with ≥ 10 eyes and minimum follow-up of 24 months. [file Table4.docx]

**Supplemental Table 4. Allogeneic SLET rejections for case series with ≥ 10 eyes and minimum follow-up of 24 months**

|  | Rejection Rate (eyes) | Signs of Rejection | Treatment |
| --- | --- | --- | --- |
| Shanbhag et al. (2019)^53^ | 2/30 (6%) | Sudden drop in vision associated with epithelial haze, positive fluorescein staining, and engorged superficial blood vessels encroaching toward the transplants. | Increasing topical steroids and pulse dose of IV methylprednisolone |
| Prabhasawat et al. (2021)^54^ | 3/17 (18%) | Not defined | Intravenous and topical methylprednisolone with a combination of oral cyclosporine and MMF |
| Riedl et al (2024)^55^ | 0/14 (0%) | Circumcorneal congestion and tortuosity of perilimbal blood vessels | Not defined |
